# Menthol in Dentistry

**Published:** 1885-08

**Authors:** C. E. H. Phillips

**Affiliations:** New York


### MENTHOL IN DENTISTRY.

BY C. E. H. PHILLIPS, D. D. S., NEW YORK.

Any addition to the list of dental remedies that assist in combatting disease and relieving pain is always desirable. Presenting some claims for consideration and trial is the "Peppermint Camphor Menthene," more generally known as "Menthol." In the March number of the INDEPENDENT PRACTITIONER appears a report of the gratifying results attending the use of a solution of menthol in cases of pericementitis and alveolar abscess, by a dental practitioner; also stating that Dr. Hungerford, a physician, had relieved an aching tooth with the same agent.

Not long since a patient presented himself at my office for a general inspection of the teeth, and directed attention to a superior bicuspid, which had given much pain. Having a menthol "pencil," the patient had endeavored to relieve the disturbance by applying the same upon the face, over the offending member, but with no result save the cold sensation produced by the volatilization of the agent. The pain having become more severe, the patient then raised the lip and applied the pencil directly to the gum. Here the sensation produced was described as warm and grateful, with a marked diminution of pain in a short time, which, after a second application, entirely ceased. I have employed the solution from the formula suggested in the journal, both simple and combined with other medicaments, with good results.

In cases of facial neuralgia, as a topical application over the nerve track, it is often highly gratifying, and particularly is this the case when the cause originates within the oral cavity, where it may at the same time be employed.

A trial of a *good* menthol preparation will place it in the medicine case of the dental practitioner as an agent of no little value.

